# Safflower Yellow Injection Alleviates Myocardial Ischemia/Reperfusion Injury by Reducing Oxidative and Endoplasmic Reticulum Stress

**DOI:** 10.3390/ph17081058

**Published:** 2024-08-12

**Authors:** Wulin Liang, Mingqian Zhang, Jiahui Gao, Rikang Huang, Lu Cheng, Liyuan Zhang, Zhishan Huang, Zhanhong Jia, Shuofeng Zhang

**Affiliations:** 1 School of Chinese Materia, Beijing University of Chinese Medicine, Beijing 102488, China20230935226@bucm.edu.cn (L.Z.);; 2 Department of Tibetan Pharmacy, University of Tibetan Medicine, Lhasa 850030, China

**Keywords:** safflower yellow injection, myocardial ischemia/reperfusion injury, apoptosis, oxidative stress, endoplasmic reticulum stress

## Abstract

Safflower yellow is an extract of the famous Chinese medicine *Carthamus tinctorious* L, and safflower yellow injection (SYI) is widely used clinically to treat angina pectoris. However, there are few studies on the anti-myocardial ischemia/reperfusion (I/R) injury effect of SYI, and its mechanisms are unclear. In the present study, we aimed to investigate the protective effect of SYI on myocardial I/R injury and explore its underlying mechanisms. Male Sprague Dawley rats were randomly divided into a control group, sham group, model group, and SYI group (20 mg/kg, femoral vein injection 1 h before modeling). The left anterior descending coronary artery was ligated to establish a myocardial I/R model. H9c2 cells were exposed to oxygen–glucose deprivation/reoxygenation (OGD/R) after incubation with 80 μg/mL SYI for 24 h. In vivo, TsTC, HE, and TUNEL staining were performed to evaluate myocardial injury and apoptosis. A kit was used to detect superoxide dismutase (SOD) and malondialdehyde (MDA) to assess oxidative stress. In vitro, flow cytometry was used to detect the reactive oxygen species (ROS) content and apoptosis rate. Protein levels were determined via Western blotting. Pretreatment with SYI significantly reduced infarct size and pathological damage in rat hearts and suppressed cardiomyocyte apoptosis in vivo and in vitro. In addition, SYI inhibited oxidative stress by increasing SOD activity and decreasing MDA content and ROS production. Myocardial I/R and OGD/R activate endoplasmic reticulum (ER) stress, as evidenced by increased expression of activating transcription factor 6 (ATF6), glucose-regulated protein 78 (GRP78), cysteinyl aspartate-specific proteinase caspase-12, and C/EBP-homologous protein (CHOP), which were all inhibited by SYI. SYI ameliorated myocardial I/R injury by attenuating apoptosis, oxidative damage, and ER stress, which revealed new mechanistic insights into its application.

## 1. Introduction

Acute myocardial infarction (AMI) is a major public health risk with high morbidity and mortality worldwide. Although timely and successful restoration of blood flow is a common and effective strategy for treating AMI that can reduce myocardial ischemic injury, limit infarct size, improve ventricular dysfunction, and reduce acute mortality, restoring blood flow can cause additional myocardial tissue damage and progressively worsen it, which is known as myocardial I/R injury [1]. Due to the potential risk of myocardial I/R injury, reperfusion is known as a double-edged sword. The pathological mechanisms of myocardial I/R injury are complex. Inflammation, calcium overload, oxidative stress, endoplasmic reticulum (ER) stress, and apoptosis are the bases of myocardial I/R injury [2].

Oxidative stress is the leading cause of myocardial I/R injury and is typically associated with elevated levels of reactive oxygen species (ROS) [3]. ROS are produced in large amounts during reperfusion, exceeding the antioxidant defense capacity of myocardial cells and causing oxidative stress, which can cause oxidative damage to proteins, lipids, and DNA, resulting in cell apoptosis and local inflammatory reactions, thereby damaging a large number of cardiomyocytes [4]. Recently, numerous studies have confirmed that oxidative stress can promote apoptosis through various mechanisms, such as activating apoptotic signaling pathways and increasing the expression of pro-apoptotic genes [5]. ER stress is another key factor that contributes to myocardial I/R injury. The main function of the ER is to participate in protein synthesis and folding, which are easily susceptible to disturbances by various pathological stimuli (e.g., oxidative stress) during myocardial I/R [6]. Disturbed ER function causes the formation of unfolded/misfolded proteins and their accumulation in the ER lumen, triggering ER stress [7]. Three transmembrane signaling proteins, activating transcription factor 6 (ATF6), protein kinase RNA-like ER kinase (PERK), and inositol-requiring enzyme−1α (IRE1α), participate in the unfolded protein response (UPR) of the ER. Under physiological conditions, these three transmembrane proteins bind to GRP78 and remain inactive [7]. When ER stress occurs, GRP78 is recruited by unfolded or misfolded proteins and dissociates from IRE1, ATF6, and PERK. After IRE1, ATF6, and PERK are activated, the UPR is initiated [8]. As an adaptive survival response, the UPR maintains cell homeostasis by increasing ER-resident chaperone proteins, accelerating unfolded protein degradation, and reducing protein synthesis. However, a prolonged and severe UPR will activate the ER stress-induced apoptosis that is mainly mediated by the C/EBP-homologous protein (CHOP) and cysteinyl aspartate-specific proteinase caspase-12 pathways [9]. Moreover, oxidative and ER stress can promote each other, interfere with cell functions, and ultimately activate apoptotic signaling [10]. Therefore, the inhibition of oxidative and ER stress is an effective therapeutic method for alleviating myocardial I/R injury.

Safflower yellow injection (SYI) is an extract of *Carthamus tinctorious* L., a well-known Chinese herb that removes blood stasis and activates blood circulation. The main active ingredient of SYI is hydroxysafflower yellow A (HSYA). It was reported that HSYA has various pharmacological effects, including anticoagulation, antithrombosis, anti-aging, anti-fatigue, antioxidant, and analgesic effects [11]. Based on the strong bioactivity of HSYA, SYI has become the main clinical drug prescribed to treat cerebrovascular and cardiovascular diseases such as stroke and myocardial infarction. However, currently, there is very little research on the efficacy and mechanism of SYI when treating myocardial I/R injury. Although a recent study demonstrated that SYI reduced myocardial I/R injury in rats by regulating the Toll-like receptor 4/nuclear factor-κB pathway to inhibit inflammation [12], the underlying mechanisms by which SYI protects against myocardial I/R injury remain largely unexplored. It is currently unclear whether SYI can improve myocardial I/R injury by regulating oxidative and ER stress. Therefore, in this study, we aimed to investigate the protective effect of SYI on myocardial I/R injury and explore its mechanisms from the perspective of anti-oxidation and anti-ER stress. Our research enhances our understanding of the pharmacological mechanism by which SYI treats myocardial I/R injury and provides strong experimental evidence for the clinical treatment of cardiovascular diseases with SYI.

## 2. Results

### 2.1. SYI Attenuated Myocardial I/R Injury in Rats

**Figure 1:**
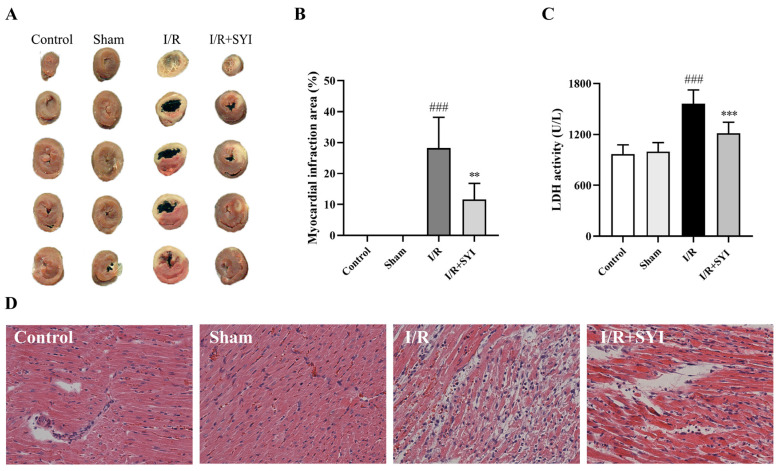
SYI reduced myocardial I/R injury in rats. (**A**) Representative photographs of TTC-stained heart slices. (**B**) Quantitative analysis of the infarct area (*n* = 6). (**C**) LDH activity in serum (*n* = 6). (**D**) HE staining shows pathological changes in the myocardium (200× magnification; scale bar is 100 μm). ^###^ *p* < 0.001 compared with the sham group. ** *p* < 0.01 and *** *p* < 0.001 compared with the model group.

We first evaluated the effects of SYI on myocardial injury caused by myocardial I/R in rats. Myocardial infarct size is a common and important indicator of the extent of I/R injury, and Triphenyltetrazolium chloride (TTC) staining is one of the most commonly used methods to detect myocardial infarct size [13]. As shown in Figure 1A, the myocardial infarct area increased by 28.23% in the model group compared with the sham group, and SYI pretreatment reduced the infarct area to 11.63%. These effects were further confirmed via quantitative analysis of the TTC staining in Figure 1B. Compared to the I/R group, the SYI pretreatment also markedly downregulated the level of LDH in the model rats (from 1563.12 U/L to 1214.00 U/L), as shown in Figure 1C, which corresponded with the TTC staining result. The pathological changes in the myocardial tissue were shown by hematoxylin–eosin (HE) staining. As shown in Figure 1D, the model group exhibited obvious myocardial injuries, such as abundant inflammatory cell infiltration, bleeding between myocardial fibers, myocardial fiber necrosis, and myocardial cell swelling. SYI could somewhat reduce pathological injuries to the myocardium in rats.

### 2.2. SYI Attenuated I/R-Induced Myocardial Apoptosis in Rats

**Figure 2:**
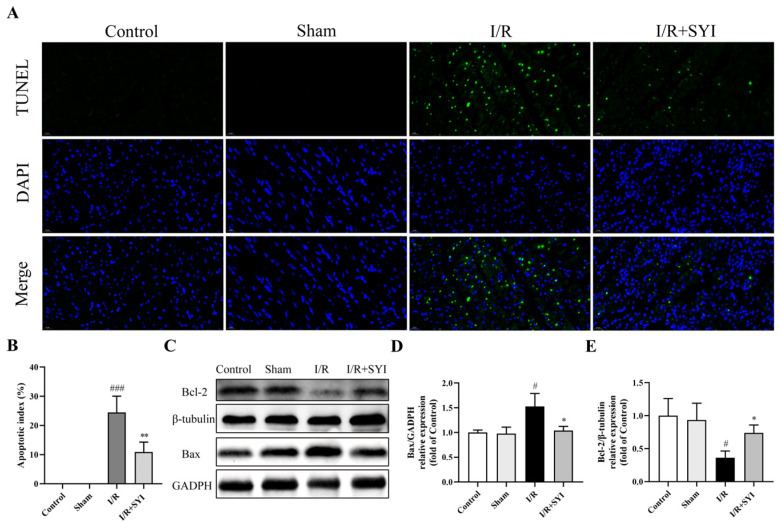
SYI attenuated I/R-induced myocardial apoptosis in rats. (**A**) Representative TUNEL staining (400× magnification; scale bar is 20 μm). (**B**) Quantitative analysis of apoptosis (*n* = 3). (**C**) The expression levels of Bax and Bcl−2 were analyzed via Western blotting. (**D**,**E**) The relative protein expression of Bax and Bcl−2 (*n* = 3). ^#^ *p* < 0.05 and ^###^ *p* < 0.001 compared with the sham group. * *p* < 0.05 and ** *p* < 0.01 compared with the model group.

The anti-apoptotic effect of SYI was detected via TdT-mediated dUTP nick-end labeling (TUNEL) staining. As shown in Figure 2A,B, in the model group, the rate of apoptotic cardiomyocytes increased to 24.5%, and pretreatment with SYI could effectively reduce the apoptotic rate to 10.9%. Moreover, Western blotting was used to examine apoptosis-related proteins. As shown in Figure 2C–E, SYI pretreatment was associated with an increase in the B-cell lymphoma-2 (Bcl-2) level and a decrease in the Bcl2-associated X (Bax) level in the myocardia of model-group rats.

### 2.3. SYI Attenuated I/R-Induced Oxidative and ER Stress in Rat Hearts

**Figure 3:**
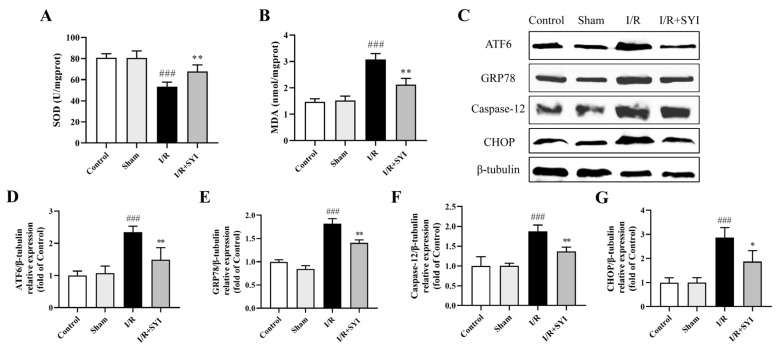
SYI attenuated I/R-induced oxidative and ER stress in rat hearts. (**A**,**B**) The SOD activity and MDA content in the myocardium (*n* = 6). (**C**) The expression levels of ER stress-related proteins (ATF6, GRP78, caspase-12, and CHOP) were analyzed via Western blotting. (**D**–**G**) The relative protein expression of ATF6, GRP78, caspase-12, and CHOP (*n* = 3). ^###^ *p* < 0.001 compared with the sham group. * *p* < 0.05 and ** *p* < 0.01 compared with the model group.

The malondialdehyde (MDA) content and superoxide dismutase (SOD) activity were determined to evaluate the antioxidation property of SYI. The results in Figure 3A,B show that myocardial I/R markedly decreased the SOD activity and increased the MDA content. However, SYI treatment could attenuate these changes. To study the effect of SYI treatment on ER stress, Western blotting was used to examine the ER stress-related proteins ATF6, GRP78, CHOP, and caspase-12. The results in Figure 3C–G show that I/R injury upregulated the expression of ATF6, GRP78, CHOP, and caspase-12 and that SYI pretreatment significantly decreased the expression levels of these proteins.

### 2.4. SYI Reduced OGD/R-Induced Injury in H9c2 Cells

**Figure 4:**
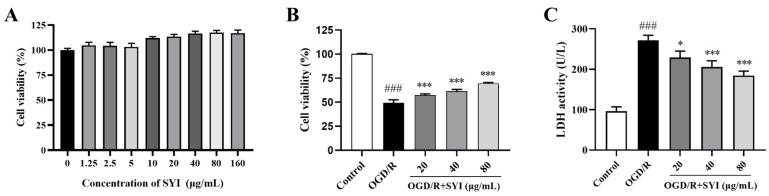
SYI reduced OGD/R-induced injury in H9c2 cells. (**A**) Cell viability of H9c2 cells incubated with different concentrations of SYI for 24 h. (**B**) Effects of SYI on cell viability after OGD/R (*n* = 6). (**C**) Effects of SYI on OGD/R-induced LDH leakage (*n* = 3). ^###^ *p* < 0.001 compared with the sham group. * *p* < 0.05 and *** *p* < 0.01 compared with the model group.

First, we evaluated the proliferative toxicity of SYI in H9c2 cells. As shown in Figure 4A, after being treated with SYI for 24 h at 1.25~160 μg/mL, the cell viability did not decrease, indicating that SYI has no proliferative toxicity at the concentrations of 1.25~160 μg/mL. Then, the OGD/R model was used to induce H9c2 cell injuries and observe the effect of SYI. As shown in Figure 4B,C, pretreatment with 20, 40, or 80 μg/mL SYI effectively increased cell viability and decreased LDH leakage. In particular, the concentration of 80 μg/mL had the best effects. Therefore, 80 μg/mL was the optimal dose to study the mechanisms by which SYI protects against OGD/R injury in the follow-up experiments.

### 2.5. SYI Suppressed OGD/R-Induced Apoptosis in H9c2 Cells

**Figure 5:**
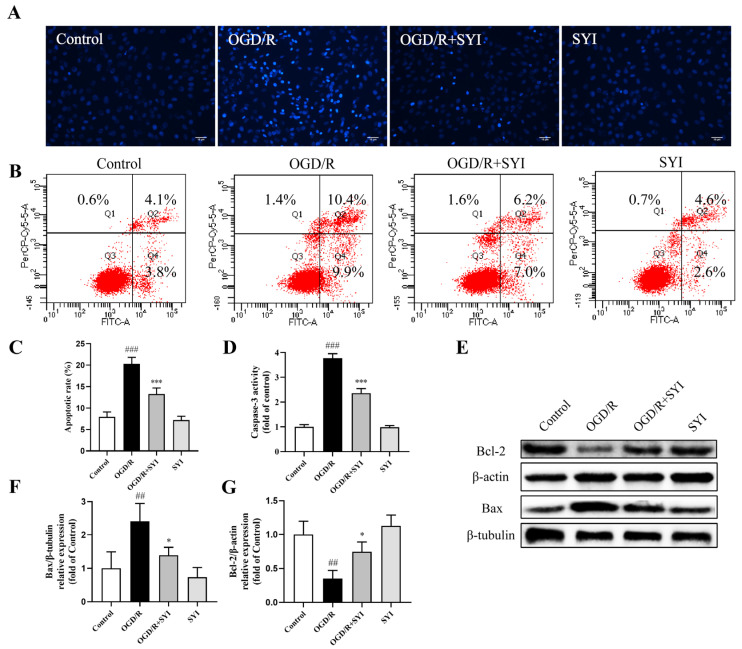
SYI reduced OGD/R-induced apoptosis in H9c2 cells. (**A**) Hoechst 33342 staining showing the anti-apoptotic potential of SYI. (**B**) Apoptosis in H9c2 cells was analyzed via flow cytometry. (**C**) Quantitative analysis of the apoptotic rate (*n* = 3). (**D**) Caspase-3 activity (*n* = 3). (**E**) The expression levels of Bax and Bcl-2 were analyzed via Western blotting. (**F**,**G**) The relative protein expression of Bax and Bcl-2 (*n* = 3). ^##^ *p* < 0.01 and ^###^ *p* < 0.001 compared with the control group. * *p* < 0.05 and *** *p* < 0.001 compared with the model group.

The Hoechst 33342 staining assay is often used to detect apoptosis, as the nuclei of apoptotic cells are densely stained. As illustrated in Figure 5A, the OGD/R group exhibited an increase in condensed or fragmented chromatin, and SYI treatment inhibited this effect, indicating its ability to inhibit apoptosis. Annexin V-FITC/PI staining was applied to further evaluate the anti-apoptosis effect of SYI. As illustrated in Figure 5B,C, the apoptotic rate of H9c2 cells increased significantly after OGD/R treatment, while incubation with SYI could inhibit OGD/R-induced apoptosis. The inhibition of apoptosis by SYI was further confirmed by detecting the expression of Bax and Bcl-2 as well as caspase-3 activity. As shown in Figure 5D–G, the OGD/R treatment increased the Bax level and caspase-3 activation and decreased the Bcl-2 level. However, SYI pretreatment reversed these changes. All of these results support the idea that SYI could reduce OGD/R-induced cardiomyocyte injury partly by suppressing apoptosis.

### 2.6. SYI Attenuated OGD/R-Induced Oxidative and ER Stress in H9c2 Cells

**Figure 6:**
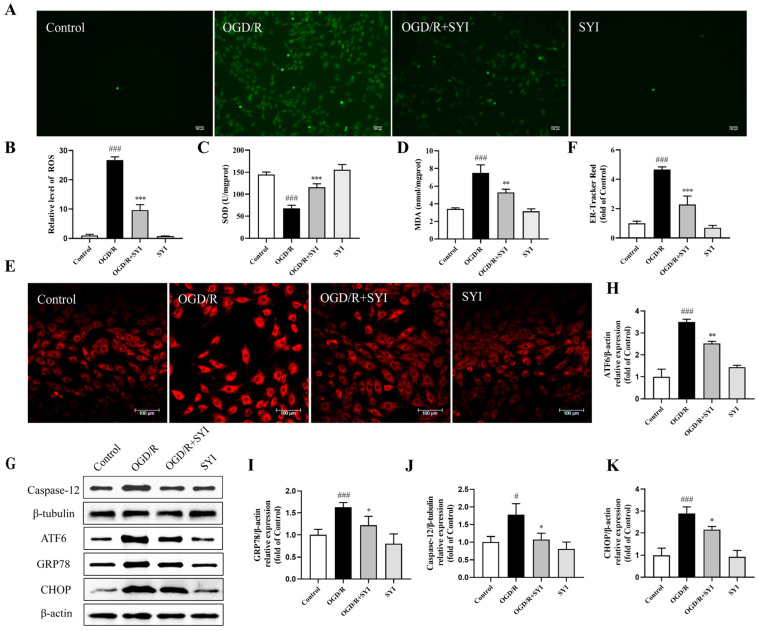
SYI attenuated OGD/R-induced oxidative and ER stress in H9c2 cells. (**A**) The ROS expression level was observed. (**B**) Quantitative analysis of ROS fluorescence intensity. (**C**,**D**) SOD activity and MDA contents in cells (*n* = 3). (**E**) The level of ER stress was assessed. (**F**) Quantitative analysis of ER-Tracker Red staining (*n* = 3). (**G**) The expression levels of ER stress-related proteins (ATF6, GRP78, caspase-12, and CHOP) were analyzed via Western blotting. (**H**–**K**) The relative protein expression of ATF6, GRP78, caspase-12, and CHOP (*n* = 3). ^#^ *p* < 0.05 and ^###^ *p* < 0.001 compared with the sham group. * *p* < 0.05, ** *p* < 0.01, and *** *p* < 0.001 compared with the model group.

The production of ROS is induced in the process of OGD/R, resulting in oxidative damage. The results in Figure 6A–D show that OGD/R treatment significantly increased the intracellular ROS and MDA levels and significantly decreased the SOD activity. However, SYI treatment could attenuate these changes, indicating that SYI could reduce OGD/R-induced cardiomyocyte injury through antioxidation. ER-Tracker Red staining was used to detect ER stress damage [14]. The results in Figure 6E,F show that the OGD/R treatment markedly increased the fluorescence intensity of the ER and that SYI reduced this effect. ER stress-related proteins were further detected to investigate whether SYI could reduce the ER stress induced by OGD/R. As illustrated in Figure 6G–K, OGD/R treatment increased ATF6, GRP78, CHOP, and caspase-12 expression, and SYI pretreatment significantly suppressed these proteins. These results suggest that ER stress is involved in OGD/R-induced injury and that SYI partly protects cardiomyocytes by resisting ER stress.

## 3. Discussion

Our study found that SYI could decrease myocardial infarct size, improve pathological changes in rats with myocardial I/R, and reduce H9c2 cell injuries induced by OGD/R. These results support SYI as a promising drug for treating myocardial I/R injury, but its mechanism of action still needs to be determined.

During myocardial I/R injury, apoptosis is the main cause of cell death [15]. In the mechanism of cell apoptosis, the Bcl-2 family plays an important role. Bcl-2 family proteins affect cell survival by regulating mitochondrial membrane permeability. Bcl-2, as an anti-apoptotic cytokine, maintains mitochondrial membrane integrity, while Bax, as a pro-apoptotic protein, promotes mitochondrial membrane permeability [16]. The Bax and Bcl-2 expression equilibrium is an important factor that determines apoptosis. Caspase-3 is a key molecule involved in apoptosis signaling, and once activated, it can irreversibly amplify the cell apoptosis signaling pathway. Therefore, caspase-3 is the executor of cell apoptosis [17]. In our study, SYI treatment effectively inhibited cardiomyocyte apoptosis, as further demonstrated by Hoechst 33342 and Annexin V FITC/PI staining. At the same time, SYI reduced the expression of the apoptosis-related proteins Bax and caspase-3, increased Bcl-2 levels to alleviate cell apoptosis, and thus inhibited myocardial I/R injury. This proved that SYI exhibited anti-apoptotic properties during myocardial I/R.

Oxidative stress plays a key role in the pathophysiological mechanism of myocardial I/R and can induce apoptosis of myocardial cells [18]. Myocardial I/R activates oxidative stress, ROS are produced in large amounts, and the activities of antioxidant enzymes are reduced, which decreases the organism’s oxidation resistance and results in oxidative damage [19]. ROS promote apoptosis by downregulating and degrading Bcl-2 through ubiquitination [20]. SOD is the first line of defense for cells to resist oxidative damage and is the main ROS scavenger [21]. ROS react with polyunsaturated fatty acids during oxidative stress to produce a series of complex compounds, including MDA [22]. The MDA content often reflects the degree of lipid peroxidation. The main effective component in SYI is HSYA, which was reported to alleviate oxidative damage to cardiomyocytes by increasing the activities of antioxidant enzymes and reducing the production of ROS [23]. This suggests that SYI has the potential to reduce oxidative stress and myocardial I/R injury. Our in vivo and in vitro observations suggest that SYI can alleviate myocardial damage caused by oxidative stress by reducing the MDA content and increasing SOD activity. Therefore, SYI has been proven to provide good antioxidant stress resistance.

Accumulating evidence has proved that ER stress-induced apoptosis is an important factor that causes myocardial I/R injury [24,25]. Excessive ROS is a pathological stimulus that contributes to ER stress during myocardial I/R. Meanwhile, ER stress also induces the generation of ROS from the ER and mitochondria. Thus, oxidative and ER stress promote each other and activate apoptosis together [26]. Under normal physiological conditions, GRP78 can bind to the three key enzymes involved in stress signal transduction in the ER (PERK, ATF6, and IRE1). When ER homeostasis is disrupted, ATF6 dissociates from GRP78, which promotes the activation of ATF6 signaling during ER stress. ATF6 signaling is a pro-apoptotic effector that induces the activation of CHOP and caspase-12 [27,28]. CHOP is the key activator of ER stress-induced apoptosis during myocardial I/R [29]. Several mechanisms are involved in CHOP-mediated apoptosis. For example, CHOP induces the expression of the death receptor DR5, leading to caspase-8 activation, thereby activating the exogenous apoptosis pathway [30]. CHOP also suppresses the expression of Bcl-2 in cardiomyocytes, promoting the release of cytochrome c from mitochondria and activating the apoptosis cascade [31]. Moreover, caspase-12 is another important activator of apoptosis and is specifically expressed during ER stress-related apoptosis [32]. Activated caspase-12 initiates apoptosis by activating caspase-9 to activate caspase-3 [33]. In the current study, SYI decreased ATF6, GRP78, CHOP, and caspase-12 levels in vivo and in vitro, suggesting that SYI could effectively inhibit ER stress-mediated apoptosis pathways during myocardial I/R. In summary, we report for the first time that SYI may improve myocardial I/R injury by regulating ER stress. In the future, it will be necessary to further verify the role of SYI in improving myocardial I/R injury by activating or inhibiting ER stress signaling both in vivo and in vitro.

We preliminarily investigated the protective effect of SYI against myocardial I/R injury in rats and explored its mechanisms from the perspective of anti-oxidation and anti-ER stress. However, this study has some limitations. For example, we did not further investigate whether the overproduction of ROS is related to mitochondrial dysfunction. We did not activate or inhibit ER stress signaling in vivo and in vitro to further validate the role of SYI. In addition, the upstream–downstream connection between SYI-modulated oxidative and ER stress needs to be further explored. A more comprehensive experimental design using gene editing, pharmacological methods, and multi-omics technology can be performed in the future to further investigate the potential mechanisms by which SYI combats myocardial I/R injury.

## 4. Materials and Methods

### 4.1. Drug and Reagents

SYI (drug approval No. Z20050146, batch No. 1810216) was provided by Zhejiang Yongning Pharmaceutical Co., Ltd. (Taizhou, China). TTC and HE staining kits were purchased from Solarbio Technology (Beijing, China). 4′, 6-diamino-2-phenylindole (DAPI); a Hoechst 33242 staining solution; an ER-tracking Red fluorescent probe; and ROS, TUNEL, MDA, SOD, and caspase-3 activity assay kits were purchased from Beyotime Biotechnology (Shanghai, China). Primary antibodies against ATF6, CHOP, GRP78, Bax, Bcl-2, GAPDH, β-tubulin, and β-actin, as well as goat anti-mouse and anti-rabbit secondary antibodies, were purchased from Protein Group Inc. (Chicago, IL, USA). A primary antibody against caspase-12 was purchased from Abcam (Cambridge, UK).

### 4.2. Animals

Adult male Sprague Dawley rats weighing 220~250 g (*n* = 96) were purchased from SPF (Beijing) Biotechnology Co., Ltd. (No. SCXK2020-0033, Beijing, China). Food and water were freely available to the animals in standard laboratory conditions with a 12 h photoperiod. Our research was approved by the Medical and Experimental Animal Ethics Committee of Beijing University of Chinese Medicine (BUCM4-2020020101-1026).

### 4.3. Establishment of Model and Drug Administration

The rats were anesthetized by intraperitoneally injecting 1% pentobarbital sodium (40 mg/kg), and they were connected to a ventilator for artificial respiration. Ischemia was caused by ligating the left anterior descending (LAD) coronary artery with a 6-0 silk suture. After 45 min of ischemia, the ligation line was removed, and reperfusion lasted 24 h. The same operation was performed on the sham group except that the LAD coronary artery was not blocked. The control group did not undergo any operation.

In our previous dose–effect relationship experiments, we found that 20 mg/kg SYI could reduce the myocardial infarct area in a rat model of myocardial I/R [34]. Therefore, in the present study, we chose the concentration of 20 mg/kg for the mechanism study. The rats were randomized into four groups (*n* = 24): a control group, a sham group, a myocardial I/R group, and an I/R + 20 mg/kg SYI group. The rats were injected with 20 mg/kg of SYI via the femoral vein 1 h before surgery, and the same amount of 0.9% saline was administered to the sham and I/R groups.

### 4.4. Measurement of Myocardial Infarct Area

After reperfusion, the hearts of the rats in each group were immediately removed and frozen in a medical refrigerator at −80 °C for 10 min. Then, the heart ventricles were cut into 5 slices with a thickness of about 1 mm. The slices were stained with 1% TTC in dark conditions for 15 min and subsequently fixed in a paraformaldehyde solution (4%) for 24 h. After the slices were fixed, a normal myocardium appeared red and an infarcted myocardium appeared white. Image J software was used to measure each slice of the myocardium’s total area and infarct area, and the percentage of infarct area in the total myocardial area was calculated.

### 4.5. HE Staining

After reperfusion, the hearts were immediately removed, the blood was rinsed off, and the hearts were fixed in a sufficient amount of a paraformaldehyde solution (4%) for 24 h. This was followed by dehydration, paraffin embedding, sectioning, dewaxing, hydration, and HE staining. Myocardial histopathology was observed, and images were obtained using an optical microscope (ECLIPSE 80i, Nikon, Tokyo, Japan).

### 4.6. Detection of Myocardial Apoptosis

After reperfusion, the hearts of the rats in each group were removed, rinsed with normal saline, and fixed in a paraformaldehyde solution (4%) at room temperature for 24 h, followed by embedding and sectioning. TUNEL staining was performed according to the manufacturer’s instructions. The DAPI reagent stained the nuclei of all cardiomyocytes blue, while the TUNEL reagent stained the nuclei of apoptotic cardiomyocytes green. The ratio of TUNEL-positive cardiomyocytes vs. total cardiomyocytes was calculated as the apoptosis index.

### 4.7. Determination of SOD Activity and MDA Content

For in vivo samples, rat hearts were removed after reperfusion, homogenized in saline, and centrifuged at 4 °C and 4000 r/min for 10 min to obtain the supernatant. For in vitro samples, cells were collected after the different treatments. The SOD activities and MDA contents in the myocardia and cells were detected according to the manufacturer’s instructions.

### 4.8. Western Blotting

Cardiomyocytes and heart tissues were collected, lysed using RIPA buffer, and centrifuged to obtain the protein supernatant. A BCA assay kit was applied to quantify the protein concentration. The proteins were separated via SDS-PAGE and transferred to PVDF membranes. The membranes were incubated with a skim milk buffer (5%) for blocking. After 1.5 h, the corresponding primary antibodies were applied for overnight incubation at 4 °C. After incubation with the secondary antibody at room temperature for 1 h, the PVDF membranes were incubated with an ECL reagent for 5 min and visualized using a Chemiluminescence Imaging System (ChemiDoc^TM^ MP, Bio-Rad, Hercules, CA, USA).

### 4.9. Cell Culture and OGD/R

H9c2 cells were obtained from the Chinese Academy of Sciences (Beijing, China). The cells were grown in high-glucose DMEM containing 10% fetal bovine serum, 1% penicillin/streptomycin, and 4 mM L-glutamine at 37 °C in a 5% CO_2_ incubator.

When the cells reached 70–80% confluence, they were incubated with 80 μg/mL SYI for 24 h before OGD/R. Then, the high-glucose DMEM was replaced with no-glucose DMEM, and the cells were transferred to a hypoxia incubator (5% CO_2_, 95% N_2_) at 37 °C. After 6 h, the medium was replaced with high-glucose DMEM, and the cells were moved to the regular incubator to be cultured for an additional 12 h.

The H9c2 cells were divided into the following groups: a control group (incubated under normoxic conditions), an OGD/R group (treated as described in the above procedures), an OGD/R + SYI group (treated with SYI for 24 h before OGD/R), and an SYI group (treated with SYI for 24 h and incubated under normoxic conditions).

### 4.10. Cell Viability Determination

The CCK-8 assay was used to determine cell viability. H9c2 cells were plated on 96-well plates (1 × 10^4^ cells per well). After the different treatments, the original medium in the wells was replaced with 10% CCK-8 diluted in a fresh medium. After incubation at 37 °C for 1 h, the absorbance value was detected at 450 nm using a microplate reader (EPOCH, Bio Tek, Winooski, VT, USA), and the cell viability was calculated.

### 4.11. Measurement of LDH

H9c2 cells were seeded on 6-well plates (2 × 10^5^ cells per well). After the different treatments, the cell supernatants were used to detect the LDH activity. All procedures were carried out according to the manufacturer’s instructions.

### 4.12. Detection of Intracellular ROS

H9c2 cells were seeded on 6-well plates (2 × 10^5^ cells per well). After the different treatments, the original medium in the wells was replaced with a serum-free medium containing 0.1% DCFH-DA. After incubation for 25 min at 37 °C, residual extracellular DCFH-DA was removed by washing the cells 3 times with a serum-free medium. Subsequently, the cells were observed and photographed using an inverted fluorescence microscope (ECLIPSE Ts2R, Nikon, Tokyo, Japan).

### 4.13. Hoechst 33342 Staining and Annexin V-FITC/PI Staining

Hoechst 33342 is a common reagent for detecting apoptosis. After the treatments, the original medium was removed and a Hoechst 33342 solution was added. After incubation with Hoechst 33342 at room temperature for 5 min in the dark, the fluorescence intensity and nuclear morphology were observed using a fluorescent microscope.

An Annexin V-FITC assay kit was used to further detect apoptosis. After the treatments, the cells were harvested and gently resuspended in an Annexin V binding buffer (195 µL) and incubated with a PI (10 µL) and Annexin V-FITC solution (5 µL) in the dark for 10 min at 37 °C. Apoptosis was detected using a flow cytometer (FACSCanto II, BD Biosciences, FL, NJ, USA). The apoptosis rate was calculated as the percentage of Annexin V-positive cells among all cells.

### 4.14. Analysis of Caspase-3 Activation

H9c2 cells were collected to detect the caspase-3 activity according to the manufacturer’s protocols. Briefly, after the cells were lysed, the supernatant was obtained via centrifugation and incubated with an Ac-DEVD-*p*NA substrate at 37 °C. The fluorescence intensity was measured at 405 nm using a microplate reader. The results are presented as the ratio of each group to the control group.

### 4.15. ER Stress Analysis

An ER-tracking Red fluorescent probe was used to detect the level of ER stress. After the treatments, the culture medium was replaced with a preheated ER-Tracker Red working solution (1 μM). After incubation for 30 min at 37 °C, the cells were washed with PBS and observed using a confocal microscope (SP8, Leica, Hessian, Germany) with excitation and emission wavelengths of 587 nm and 615 nm.

### 4.16. Statistical Analysis

GraphPad Prism 8.0.1 software was used for statistical processing, and the data for each group were expressed as means ± standard deviations (S.D.). When the sample data did not conform to a normal distribution, nonparametric tests were used. When the data conformed to a normal distribution, a one-way ANOVA was used, and groups were compared using the LSD method. Significant differences were identified at *p* < 0.05.

## 5. Conclusions

We demonstrated the protective effects of SYI against myocardial I/R injury and confirmed that it inhibited apoptosis. This was largely due to the suppression of oxidative and ER stress. These results provide an experimental basis for the clinical application of SYI and new mechanistic insights for follow-up studies on SYI. However, the mechanism of SYI’s cardioprotective action needs further study.

## Data Availability

The data are contained within this article.
